# Assessing the accuracy of an inter-institutional automated patient-specific health problem list

**DOI:** 10.1186/1472-6947-10-10

**Published:** 2010-02-23

**Authors:** Lise Poissant, Laurel Taylor, Allen Huang, Robyn Tamblyn

**Affiliations:** 1 Centre for Interdisciplinary Research in Rehabilitation of Greater Montreal, Montreal, Qc, Canada; 2 School of Rehabilitation, Faculty of Medicine, University of Montreal, Montreal, Quebec, Canada; 3 Department of Medicine, McGill University, Montreal, Quebec, Canada; 4 Department of Neurology and Neurosurgery, McGill University, Montreal, Quebec, Canada; 5 Department of Epidemiology & Biostatistics, McGill University, Montreal, Quebec, Canada

## Abstract

**Background:**

Health problem lists are a key component of electronic health records and are instrumental in the development of decision-support systems that encourage best practices and optimal patient safety. Most health problem lists require initial clinical information to be entered manually and few integrate information across care providers and institutions. This study assesses the accuracy of a novel approach to create an inter-institutional automated health problem list in a computerized medical record (MOXXI) that integrates three sources of information for an individual patient: diagnostic codes from medical services claims from all treating physicians, therapeutic indications from electronic prescriptions, and single-indication drugs.

**Methods:**

Data for this study were obtained from 121 general practitioners and all medical services provided for 22,248 of their patients. At the opening of a patient's file, all health problems detected through medical service utilization or single-indication drug use were flagged to the physician in the MOXXI system. Each new arising health problem were presented as 'potential' and physicians were prompted to specify if the health problem was valid (Y) or not (N) or if they preferred to reassess its validity at a later time.

**Results:**

A total of 263,527 health problems, representing 891 unique problems, were identified for the group of 22,248 patients. Medical services claims contributed to the majority of problems identified (77%), followed by therapeutic indications from electronic prescriptions (14%), and single-indication drugs (9%). Physicians actively chose to assess 41.7% (n = 106,950) of health problems. Overall, 73% of the problems assessed were considered valid; 42% originated from medical service diagnostic codes, 11% from single indication drugs, and 47% from prescription indications. Twelve percent of problems identified through other treating physicians were considered valid compared to 28% identified through study physician claims.

**Conclusion:**

Automation of an inter-institutional problem list added over half of all validated problems to the health problem list of which 12% were generated by conditions treated by other physicians. Automating the integration of existing information sources provides timely access to accurate and relevant health problem information. It may also accelerate the uptake and use of electronic medical record systems.

## Background

In its second report 'Crossing the Quality Chasm'[1], the Institute of Medicine identified patient safety and free flow of information as key issues to improve care. Functionalities of electronic health records (EHR) such as electronic prescribing[2-6], clinical decision support systems[7] and automated reminders [8] have been shown to be effective in improving patient safety and chronic disease management. Timely access to accurate and complete information on a person's health problems or diseases is critical to detecting drug interactions [9], preventing prescribing problems [10] and developing decision support systems based on disease-specific guidelines [11,12]. Indeed, diseases or health problems have been shown to be involved in drug interactions in more than 20% of patients in an emergency department [9] and in 6.5% of prescribing problems generated by family physicians[13]. Thus, health problem lists that are coded to enable automated surveillance and decision-support [13,14] are a key component of the Electronic Medical Record (EMR) and are instrumental in the development of decision systems that encourage best practices and optimal patient safety.

Despite the clinical value of computerized health problem lists, their adoption and sustained utilization remain low [15-17]. One of the main barriers to utilization is that computerized health problem lists require initial manual entry and active, on-going maintenance, two activities generally perceived as time consuming by physicians. Moreover, in a survey of 250 community-based physicians, Smith et al [18] identified that 13.6% of visits had missing clinical information, that the likelihood of missing information was much higher in patients with multiple health problems, and, that in more than 50% of visits, the missing information originated from outside their clinical system. Surveyed physicians perceived that the incomplete clinical information delayed the care process and could potentially lead to preventable adverse events.

Secure and timely information flow on health problems across care providers and institutions can enable safe, timely, effective, and efficient care delivery. Innovative approaches are needed that will minimize the physician's burden to manually enter diagnostic information, and access health problems identified by treating physicians in other clinics and hospital institutions. By reducing barriers to the creation of complete and up to date computerized health problem lists in electronic medical records, higher rates of EMR utilization may be forthcoming [19,20].

To date, developments in automating health problem lists have been limited to institution-specific systems with little capacity to share this information with other care providers. This lack of integration requires redundant entry of information by multiple dispersed physicians caring for the same patient, and reliance on patients' self-reported drug and disease histories that have poor accuracy [12,16,21,22]. Higher EMR utilization rates can potentially result in improved patient safety and quality of care.

This study investigated an innovative method to create an inter-institutional patient-specific health problem list for ambulatory patients using integrated administrative and electronic medical record information. The study estimated the positive predictive value of using patient-specific information from provincial health administrative diagnostic codes from medical services claims; therapeutic indications from electronic prescriptions; and single-indication drugs to generate an inter-institutional problem list, according to type and source of information.

## Methods

### The context

This study was conducted in the Canadian province of Quebec where all 7.5 million residents have access to a public health insurance program that covers the costs of all required medical care and the costs of drugs for about 50% of the population http://www.ramq.gouv.qc.ca. Each Quebec resident is assigned a unique provincial health number that can be used to link information from different data sources. The Quebec health insurance board (RAMQ) maintains a real-time online administrative health services database of all medications dispensed to Quebec beneficiaries that are covered by the RAMQ drug insurance plan and bi-weekly updates of records of all medical services claims billed for Quebec residents by all physicians in the province. Each medical claim includes the patient's unique health number, physician unique billing number (identifier), service, date, location, procedure, and an ICD-9 diagnostic code. At the time of the study, the basic ICD-9 four-digit version was used by the RAMQ.

The MOXXI automated health problem list

### Overview

The Medical Office of the XXIst century (MOXXI) system is a 'light' EMR, that includes drugs and disease management and an electronic prescriber [10,23]. Detailed information on all of the system's functionalities have been described elsewhere [10,23] and an overview of the system is available at http://moxxi.mcgill.ca. Key functionalities include; electronic prescribing, access to a patient's current drug therapy and medication history for the past 12 months, hospitalizations and emergency department visits through direct linkage to provincial databases, automated alerts for prescribing errors, stop and change prescription orders, documented allergies and management of the automated health problem list.

Three sources of information are used to generate an automated health problem list for each patient: diagnostic codes from medical services claims, health problems derived from the dispensation of single-indication drugs and therapeutic indications recorded from MOXXI-generated electronic prescriptions. Daily updates of new medical services and their respective ICD-9 codes are retrieved from the RAMQ for each consenting patient. ICD-9 codes are mapped to a commercial disease/drug knowledge database http://www.vigilance.ca to display standardized, and clinically useful French and English labels for health problems in the patient record. For example, ICD-9 codes for 'essential hypertension' and 'benign essential hypertension' are mapped to the commercial database to display 'hypertension' in the health problem list. Health problems that are identified from more than one source are assigned to one category; ordered by the following hierarchy; health problems derived from single indication dispensed drugs, medical services claims diagnostic codes and therapeutic indications from electronic prescriptions. Synonymous descriptors (e.g. hyperlipidemia and dyslipidemia) are presented to the physician who can confirm/reject the label of his choice.

An expert committee comprised of a geriatrician, clinical pharmacologist, internist and pharmacist defined 241 single-indication drugs; drugs that are each mainly used to treat one type of health problem (e.g. insulin → diabetes). Overall, 95 different health problems were linked to the 241 single-indication drugs, excluding off-label medication. Using daily updates of RAMQ prescription claims records, all dispensed single-indication drugs for each consenting patient are mapped to their corresponding health problem.

When writing prescriptions with the MOXXI system, physicians are required to document at least one therapeutic indication using a drop-down list of approved on-label and common off-label indications for that drug. Alternatively physicians can enter a free-text indication of their choice. Therapeutic indications are retrieved from each electronic prescription, mapped to the disease/drug knowledge database, and included in the health problem list.

Information from all sources is integrated into the MOXXI automated health problem list using the patient's health number as a unique identifier.

### Management of health problems

**Figure 1:**
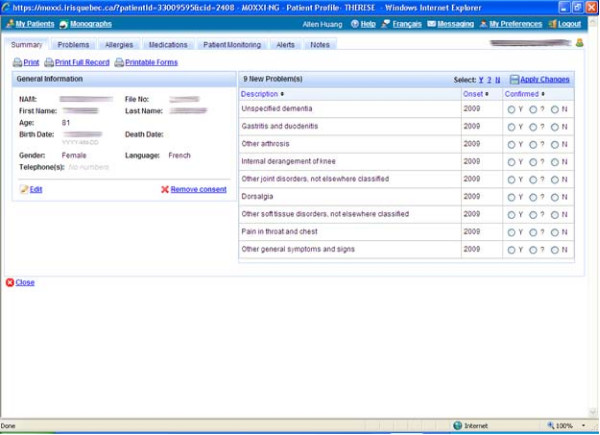
**Automated pop-up window at the opening of a patient file in the MOXXI system**.

Every time the patient's file is accessed, any new information is displayed in a pop-up window. Each new health problem record is indicated as 'potential' and physicians are prompted to record whether the health problem is valid (Y) or not (N) (Figure 1). Physicians are allowed to indicate as 'not valid' health problem they consider incorrect or resolved. Similarly, they may indicate as 'valid' health problems that are active or that are resolved but they wish to keep track of for monitoring purposes (e.g. recurrent otitis).. Health problems that are recorded as being 'not valid' are removed from the patient's visible health problem list. Records that are bypassed remain as 'potential' and continue to re-appear in the reminder pop-up window until its validity is assessed. All therapeutic indications documented by a physician while using the MOXXI prescriber are automatically included as valid health problems. However, these entries can also be subsequently removed, if they are no longer relevant. Finally, physicians can also manually add other health problems using a drop-down list from the disease/drug knowledge database or free-text entry. The physician can consult the problem list at any time, which displays all confirmed and potential problems. The health problem list is physician-specific, i.e. a health problem confirmed by one physician would appear only as a potential problem when some other physician accessed a shared patient record. The second physician can manage the shared problem list records (accept, reject, add) and an audit trail captures the changes documented in the MOXXI system.

### Evaluation of accuracy of the automated health problem list

#### Study Population

The accuracy of the automated health problem list was assessed among 121 primary care physicians who were enrolled in the MOXXI research program. Details concerning the recruitment and characteristics of the participating physicians has been described elsewhere [24]. Participating physicians were in full time fee-for-service practice in private clinics in two large metropolitan areas and were mostly men (53%). Physicians were responsible for recruiting and obtaining written consent for patients from their practice population. Data for this study were obtained from patients who consented between May 2004 and May 2007. The study was approved by McGill University Research Ethics Board, the Quebec privacy commission and the RAMQ legal counsel.

#### Data analysis

The frequency distribution of problems was estimated by source and validation status. The positive predictive value was calculated for all patients presenting problems for which the physician made an assessment. The positive predictive value was defined as the number of patients with a problem judged as being valid by the treating physician divided by the number of patients for whom the problem was identified and assessed. Positive predictive value was calculated for each problem by data source. We also estimated positive predictive value by originating physician; treating physician versus any other physician. 95% confidence intervals were estimated using the approximation method for binomial proportions. Logistic regression within a generalized estimating equation framework was used to determine if the source of diagnostic information (study physicians versus other physicians) influenced the likelihood of: 1) assessing patient's problems and 2) the validity of the information provided. Two models were fit, one for each outcome using problem as the unit of analysis, patient as the clustering variable, and an exchangeable correlation structure to account for correlation among the residuals. All data analyses were performed using SAS version 9.1 (SAS Institute, Cary, North Carolina).

## Results

**Figure 2:**
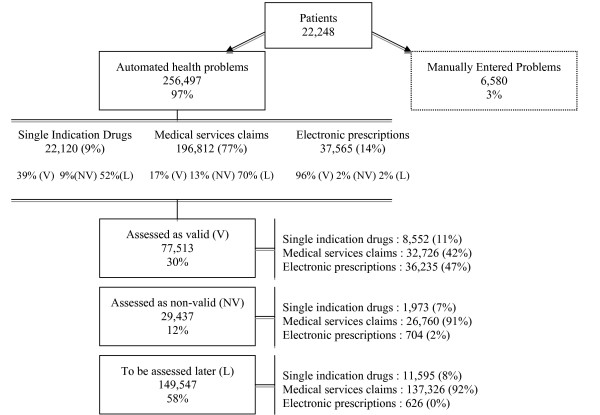
**Distribution of health problems by originating source and physicians' responses**.

In total, 22,248 patients consented to participate in the 3 year study period. Overall, 62% were female, with a mean age of 57.8 years (SD:17.7) (Table 1). A total of 263,527 health problems, representing 891 unique problems, were identified for this group of patients in the 3 year study period; an average of 5.2 health problems per patient. Each patient was dispensed, on a yearly average, 3.8 (SD: 5.6) medications of which 66% were prescribed by the study physician (Table 1). Of all health problems 256,497 (97.2%) were generated automatically by the system (Figure 2) and physicians manually entered only 6,580. All manually entered health problems were considered valid and were excluded from our analyses. Medical services claims data generated the majority (n = 196,812; 76.7%) of automated health problem records, followed by therapeutic indications from MOXXI electronic prescription records (n = 37,565; 14.7%), and then health problems derived from single-indication dispensed drug records (n = 22,120; 8.6%). Physicians assessed the validity of 41.7% (n = 106,950; 77,513 + 29,437) of automated health problems (Figure 2). The great proportion (96%) of health problems coming from electronic prescriptions were considered valid in comparison to problems derived from single-indication dispensed drugs (39%) and medical services claims (17%). Similarly, fewer health problems originating from electronic prescribing were considered non-valid (2%) followed by problems derived from single-indication dispensed drugs (9%) and medical services claims (13%).

**Table 1:** Characteristics of the 22,248 consented patients during the study period (May, 2004-May, 2007)

Characteristics	N	%
Sex		
Female	13734	62
Male	8514	38
	**Mean(SD)**	**Range**
Age (years)	57.8 (17.7)	8 - 97
Family income (CAD)	$50,289	$13,093 - $349,609
Number of confirmed health problems	5.2 (4.1)	1 - 41
Number of visits to all physicians/year^1^	10.1(13.2)	0--462
Number of visits to Study Physicians/year	3.7 (3.3)	0--61
Number of Electronic Prescriptions from Study Physician/year	1.7 (3.2)	0--115
Number of Dispensed Medications from All Physicians/year	3.8 (5.6)	0-69
Number of Dispensed Medications from Study Physicians/year	2.5 (4.1)	0-53

^1^Includes study physicians and non-study physicians seen by patients

Among all health problems that were assessed by the physicians, 29,437 (28.0%) were considered invalid at the time of the visit (Figure 2). The most frequently rejected problems from medical services claims were abscess (3.1%), dermatitis (2.3%) and aneurysm (1.9%). Hyperlipidemia (21%) and pain (16%) were the most commonly rejected problems from those derived from single-indication dispensed drugs data (Table 2).

**Table 2:** Positive predictive values of most prevalent^1 ^health problems assessed for each originating data source

Originating Source of assessed health problems	Physician's Response			
	**Considered valid** **(n)**	**Considered invalid** **(n)**	**Positive Predictive Value**	**95% Confidence Interval**

**Medical Services Claims (n = 59,486)**				
Hypertension	4767	259	95	94.2 - 95.5
Depression	1867	134	93	92.2 - 94.4
Anxiety	1330	106	93	91.3 - 94.0
Asthma	1064	240	82	79.5 - 83.7
Angina Pectoris	979	218	82	79.6 - 84.0
Osteoporosis	945	27	97	96.2 - 98.3
Diabetes, type 1	807	11	99	97.9 - 99.4
Bronchitis	778	264	75	72.0 - 77.3
Osteoarthritis	752	3	100	--
Dyslipidemia	741	24	97	95.6 - 98.1
Hypercholesterolemia	621	120	84	81.1 - 86.5
**Total Top 11 Claims (n = 17,054; 29%)**				
				
**Single Indication Drugs (n = 10,525)**				
Hyperlipidemia	1862	404	82	80.6 - 83.8
Hypothyroidism	1378	25	98	97.5 - 98.9
Diabetes Type 2	1232	52	96	94.9 - 97.0
Hypertension	1033	24	98	96.8 - 98.6
Pain	601	333	64	61.3 - 67.4
Insomnia	444	45	91	88.2 - 93.4
Iron deficiency anemia	313	116	73	68.8 - 77.2
Open angle glaucoma	198	133	60	54.5 - 65.1
Depression	185	35	84	79.3 - 88.9
Pernicious anemia	140	39	78	72.2 - 84.3
Hypokalemia	135	73	65	58.4 - 71.4
**Total Top 11 SID (n = 9,689; 92%)**				
				
**Electronic Prescribing (n = 36,939)**				
Insomnia	1991	15	99	98.9 - 99.6
Dyslipidemia	1969	19	99	98.6 - 99.5
Pain	1769	86	95	94.4 - 96.3
Hypercholesterolemia	1499	25	98	97.7 - 99.0
Hypertension	1159	2	100	--
Gastritis	916	8	99	98.5 - 99.7
Osteoarthritis	814	2	100	--
Gastroesophageal reflux	813	1	100	--
Anxiety	810	4	100	--
Angina pectoris	740	7	99	98.4 - 99.8
Allergic rhinitis	735	3	100	--
**Total Top 11 Elec.Presc. (n = 14,476; 39%)**				

^1 ^Excludes problems to be assessed later

Among problems assessed by physicians, the majority had a positive predictive value greater than 80% (Table 2). Among common problems identified by medical services claims, the positive predictive value ranged from a high of 100% (osteoarthritis) to a low of 75% (bronchitis). The positive predictive values for the most common problems identified through problems derived from single-indication dispensed drugs was within an equivalent range of a low of 60% for open angle glaucoma to a high of 98% for hypothyroidism and hypertension. Most problems identified by the therapeutic indication had positive predictive values close to 100%.

Hypertension, depression and anxiety were most often automated into patient's records from medical services claims as the originating source (Table 3). Problems derived from single-indication dispensed drugs were useful in identifying patients with hyperlipidemia, hypothyroidism and diabetes , while the MOXXI prescription therapeutic indication record was particularly useful in identifying dyslipidemia, insomnia and pain. Hypertension and hyperlipidemia were the problems most frequently presented to physicians as potential ones through the automated retrieval of information from the medical services claims and drug insurance databases, respectively. They were also the most commonly confirmed health problem (Table 3).

**Table 3:** Frequency distribution of the 20 most frequently confirmed health problems and their originating data source.

	Originating data source			
**Health problems**	**Overall Prevalence** **n (%)**	**Medical Services Claims** **n (%)**	**Single Indication** **Drugs** **n (%)**	**Electronic Prescriptions** **n (%)**

Hypertension	6889(16.7)	4697 (30.1)	1033 (15.3)	1159 (6.1)
Dyslipidemia	2604 (6.3)	635 (4.1)	0	1969 (10.3)
Depression	2437 (5.9)	1825 (11.7)	185 (2.8)	427 (2.2)
Insomnia	2435 (5.9)	0	444 (6.6)	1991 (10.5)
Hypothyroidism	2418 (5.8)	527 (3.4)	1378 (20.5)	513 (2.7)
Hyperlipidemia	2392 (5.8)	319 (2.0)	1862 (27.6)	211 (1.1)
Pain	2370 (5.7)	0	601 (8.9)	1769 (9.3)
Prevention	2296 (5.5)	0	0	2296 (12.1)
Anxiety	2095 (5.1)	1285 (8.2)	0	810 (4.3)
Diabetes type 2	2090 (5.1)	792 (5.1)	1232 (18.3)	66 (0.3)
Hypercholesterolemia	2082 (5.0)	583 (3.7)	0	1499 (7.9)
Asthma	1595 (3.9)	923 (5.9)	0	1499 (7.9)
Angina pectoris	1533 (3.8)	813 (5.2)	0	740 (3.9)
Osteoporosis	1503 (3.6)	868 (5.6)	0	635 (3.3)
Osteoarthritis	1453 (3.5)	639 (4.1)	0	814 (4.3)
Bronchitis	1191 (2.9)	726 (4.6)	0	465 (2.4)
Gastroeosophageal reflux	1114 (2.7)	301 (1.9)	0	813 (4.3)
Allergic rhinitis	1047 (2.5)	311 (2.0)	1 (0.01)	735 (3.9)
Gastritis	916 (2.2)	0	0	916 (4.8)
Eczema	891 (2.2)	374 (2.4)	0	517 (2.7)

Total Top 20 (confirmed)	41,351 (53.6%)	15,618	6,736	19,844
Total Overall (confirmed)	77,153 (100%)	32,726	8552	36,235

We examined how study physicians responded to health problem information created from medical services they provided or single indication drugs they prescribed compared to health problem information created by other physicians (Table 4). Health problems created from therapeutic indications in electronic prescriptions as well as those manually entered, were not considered in this analysis since we considered this information as 'pre-validated'. Health problems (n = 1,849; <1%) for which we could not identify the originating physician (i.e. the one who prescribed the single indication drug or claimed medical services) were excluded. Among the remaining 217,083 health problems, 91,216 (42%) originated from the physician's own practice. Our data show that, overall, study physicians were more likely to assess the validity of health problems (confirm or reject health problems) originating from their own practice (n = 35,037; 9,430 + 25,607 (38.4%)) than health problems recorded by other physicians (n = 34,098; 18,787,+ 15,311 (27.1%); p < 0.0001). Similarly, when assessed, problems originating from study physicians were significantly more likely to be valid than problems documented by other physicians (28.1% vs 12.2%, p < 0.0001).

**Table 4:** Responses of study physicians to health problems created from medical services they provided or single indication drugs they prescribed^1 ^in comparison to health problem information created by other physicians.

**Originating data source**	**Problems assessed as invalid** **n (%)**	**Problems assessed as valid** **n (%)**	**Problems not yet assessed^2^** **n (%)**	**Total** **N (%)**
Study physician	9,430 (10.3%)	25,607 (28.1%)	56,179 (61.6%)	91,216 (100%)
Other physician	18,787 (14.9%)	15,311 (12.2%)	91,769 (72.9%)	125,867 (100%)
Total	28,217 (13.0%)	40,918 (18.9%)	147,948 (68.1%)	217,083^2,3^

^1 ^Excludes electronic prescriptions (n = 37,565)

^2 ^Excludes manually entered health problems (n = 6,580) and health problems with missing physician data source (n = 1,849)

^3 ^Includes problems that may not have been seen by physicians

## Discussion

The objectives of this study were to assess the feasibility of integrating inter-institutional provincial administrative data and local clinical data to generate accurate patient-specific health problem lists. Our study showed that the majority of health problem records were generated automatically through linkages with administrative data sources, namely medical services claims (77%) and dispensed drug databases (9%). The majority of these auto-generated health problems were confirmed as valid after assessment by physicians (72.5%). When health problems that were generated and pre-validated through the electronic prescriptions (14%) are excluded, the proportion of valid health problems drops to 59%. Natural language processing (NLP), applied to progress notes, clinical reports, discharge summaries and other clinical documents has been shown to produce highly accurate health problem lists. In his study, Meystre [25] found that, among problems identified by NLP and modified by physicians, 55% of these were considered active or inactive, which is similar to the rate of validated problems found in our study. Therefore the MOXXI system can provide an alternative process to automatically generate accurate health problem lists.

The MOXXI automated problem list module was accurate in displaying the presence of chronic diseases such as hypertension, diabetes, and osteoarthritis. Even if we assume that family physicians would be aware of the patient's chronic health conditions, the automated process of retrieval through the integration of data from medical service claims and prescriptions remains more time efficient than manual entry [23]. Our study population had an average of 5.2 confirmed health problems in their problem list indicating that the automated identification of chronic and episodic illness can improve the comprehensiveness of the problem list, documenting diseases that can be used to detect drug-disease interactions at the time of electronic prescribing, and enable disease-based decision support tools.

We hypothesize that many of the health problems that were assessed as being invalid by physicians had resolved (e.g. pain) or were no longer considered clinically relevant. However, this was not true for hyperlipidemia, which was rejected 21% of the time, despite the exclusive association of certain drugs to this condition and the fact that it was the problem most often generated through the problems derived from single-indication dispensed drug data. The profile of these patients showed that 43% of them had a confirmed problem of hypercholesterolemia and 42% a confirmed problem of dyslipidemia, suggesting that physicians discarded problems which did not add further value in the management of their patients. Variation in the specificity of billing codes for certain conditions were likely responsible for a high rate of rejection, particularly when labelled by other physicians. For example "Ventricular arrhythmia" was rejected in patients whose problem lists also included: arrhythmia (34%), supraventricular arrhythmia (22%) and atrial fibrillation (17%). This action reflects the physicians' ability to filter meaningful information from the data. Because "ventricular arrhythmia" solely originated from the medical services claims data source, a coding error may have been responsible for the automated appearance of that problem [26,27]. The variable granularity of ICD-9 coding may also explain the generation of synonymous health problems, thereby inflating for some patients, the number of health problems automated by the system and the number of those that were rejected.

Although ICD-9 coding has been criticized as lacking flexibility and being too granular for immediate use by clinicians [17,28], studies have shown that most of the information physicians document within electronic health problem lists can be successfully captured by this coding scheme. Our results show that 891 ICD-9 diagnostic codes were necessary to describe the 263,077 health problems of our study population. Wilton et al [29] showed that 328 ICD-9 diagnoses accounted for 82% of health problems reported by over 3000 patients. Scherpbier [30] estimated that 36 ICD-9 diagnoses were sufficient to capture 73.4% of discharge diagnoses from a surgical unit. In our study, the 20 most prevalent diagnostics codes accounted for 53.6% of confirmed health problems for the 22,206 patients. Providing physicians with a short list of the 20 to 40 most prevalent diagnoses could facilitate the improved manual entry of an initial problem list. However, for an integrated system such as MOXXI, more than 80% of a person's health problems can be retrieved and presented to physicians automatically in a timely fashion, providing a more efficient solution for building a comprehensive problem list using existing, available and non-physician or institution specific data.

Our results show that health problems that originated from the therapeutic intent documented at the time of electronic prescribing using the MOXXI system contributed significantly to the maintenance of a patient's health problem list. More interestingly, this feature of the MOXXI system, allowed documentation of health problems, such as insomnia and dyslipidemia, which are less likely to be found in the medical services claims data or problems derived from single-indication dispensed drug databases. Thus, benefits of this additional data source were both quantitative and qualitative.

In our study, physicians tended to validate, and to keep in the health problems list, those problems, whether transient or chronic, that require ongoing care or drug management. We hypothesized that transient problems (e.g. pain) inactive and resolved issues were more likely to be discarded. Validating these hypotheses was beyond this study but our conclusions remain unchanged; optimal systems should provide clinicians the possibility of distinguishing chronic, resolved, and transient health problems from active ones, yet few systems offer this attribute [14,28,31]. At the time this study was conducted, physicians had the capacity to characterize health problems as: confirmed valid, confirmed invalid or to be reassessed later. Manual data entry was used to document any additional information. Half of the study physicians manually entered data which accounted for less than 3% (n = 6,580) of all health problems recorded. We examined the nature of manually entered health problems and found that physicians commonly recorded past medical events (more than a year prior to patient study entry) or suspected health problems. This further supports the need to offer physicians electronic problem lists that are flexible in allowing them to use it as a repertoire of all health-related information considered relevant and important to clinical care decision-making. The latest version of the MOXXI system (2009) provides them with the ability to add the attributes of time of onset, status and type of problem (medical condition, surgical procedure, disabilities, lifestyle, life events). These enhanced capabilities of the problem list will offer further capacity to develop functionalities such as automated reminders for preventive care, follow-up tests, or treatment.

Although the MOXXI system hides the data source from physicians, it was interesting to note that they were more likely to assess and validate records that originated from their own practice in comparison to records originating from claims data or visits to or dispensed drugs generated by others. This trend was evident for both medical services diagnostic codes as well as prescriptions by other physicians of single indication drugs. It was beyond the scope of this study to understand why physicians would be more likely to validate data generated from their own billing and prescriptions but time constraints and perceived responsibility or competency over certain health problems as the family physician, are possible explanations.

Our study has a number of limitations. The sensitivity of the system in identifying all health problems for each patient could not be assessed. Consequently, the capacity of the MOXXI system to provide complete information on a person's health problem is an important area for future research. Conducting an extensive chart review of the consenting patients would be needed to identify all missing health problems. However, the small proportion (2.8%) of health problems manually entered by physicians offers some indication on the completeness of the information provided by the MOXXI system. We recognize that 58% of health problems had a status of 'to be assessed later' and had to be removed from our analyses. Although our study population included patients who had at least one visit to their physician during the study period, it was not possible to identify whether the problems which remain as "potential" had been seen.

## Conclusions

Accurate and meaningful information on an individual's health is essential for optimal clinical decision-making and care delivery. Over the past two decades, the rapid expansion of information technologies in the health care system has included the development of systems aimed at providing clinicians with timely access to a person's health problem information. Despite major advances, most systems remain underutilized and the great majority of health problem lists do not have a built-in capacity to integrate health information from different data sources inside and outside a physician's practice. The automated inter-institutional health problem list function in the MOXXI system provides a mechanism to access disparate external electronic health administrative data, generate a dynamic list and to easily manage this data to produce a validated set of records in this "light" electronic medical record. The capacity to provide general practitioners a valid, complete and accurate list of a person's health problems may improve the persistent use of an EMR and its potential to improve the quality of healthcare delivery. Research is currently underway to expand the problem list to include structured information on past surgical procedures, disabilities, lifestyle and life events.

## Competing interests

The authors declare that they have no competing interests.

## Authors' contributions

LP drafted the manuscript and conceived the study. LT helped draft the manuscript. AH participated in the design of the study and the draft of the study. RT conceived of the study, its design, oversaw its coordination and helped to draft the manuscript. All authors read and approved the final manuscript.

## Pre-publication history

The pre-publication history for this paper can be accessed here:

http://www.biomedcentral.com/1472-6947/10/10/prepub
